# Intrapopulation foraging niche variation between phenotypes and genotypes of Spirit bear populations

**DOI:** 10.1002/ece3.7276

**Published:** 2021-04-13

**Authors:** Christina N. Service, Travis Ingram, Thomas E. Reimchen, Chris T. Darimont

**Affiliations:** ^1^ Department of Geography University of Victoria Victoria BC Canada; ^2^ Raincoast Conservation Foundation Sidney BC Canada; ^3^ Kitasoo Xai'xais Stewardship Authority Kitasoo/Xai'xais First Nation Klemtu BC Canada; ^4^ Department of Zoology University of Otago Dunedin New Zealand; ^5^ Department of Biology University of Victoria Victoria BC Canada

**Keywords:** multiniche polymorphism, niche diversity, Spirit bear, *Ursus*

## Abstract

Foraging niche variation within a species can contribute to the maintenance of phenotypic diversity. The multiniche model posits that phenotypes occupying different niches can contribute to the maintenance of balanced polymorphisms. Using coastal populations of black bears (*Ursus americanus kermodei)* from British Columbia, Canada, we examined potential foraging niche divergence between phenotypes (black and white “Spirit” coat color) and between genotypes (black‐coated homozygote and heterozygous). We applied the Bayesian multivariate models, with biotracers of diet (δ^13^C and δ^15^N) together comprising the response variable, to draw inference about foraging niche variation. Variance–covariance matrices from multivariate linear mixed‐effect models were visualized as the Bayesian standard ellipses in δ^13^C and δ^15^N isotopic space to assess potential seasonal and annual niche variation between phenotypes and genotypes. We did not detect a difference in annual isotopic foraging niche area in comparisons between genotypes or phenotypes. Consistent with previous field experimental and isotopic analyses, however, we found that white phenotype Spirit bears were modestly more enriched in δ^15^N during the fall foraging season, though with our modest sample sizes these results were not significant. Although also not statistically significant, variation in isotopic niches between genotypes revealed that heterozygotes were moderately more enriched in δ^13^C along hair segments grown during fall foraging compared with black‐coated homozygotes. To the extent to which the pattern of elevated δ^15^N and δ^13^C may signal the consumption of salmon (*Oncorhynchus* spp.), as well as the influence of salmon consumption on reproductive fitness, these results suggest that black‐coated heterozygotes could have a minor selective advantage in the fall compared with black‐coated homozygotes. More broadly, our multivariate approach, coupled with knowledge of genetic variation underlying a polymorphic trait, provides new insight into the potential role of a multiniche mechanism in maintaining this rare morph of conservation priority in Canada's Great Bear Rainforest and could offer new understanding into polymorphisms in other systems.

## Graphical abstract

Using coastal populations of black bears (*Ursus americanus kermodei*) from British Columbia, Canada, we examined potential foraging niche divergence between phenotypes (black and white “Spirit” coat color) and between black‐coated homozygote and heterozygous genotypes. Consistent with previous field experimental and isotopic analyses, however, we found that white phenotype Spirit bears were modestly more enriched in δ^15^N during the fall foraging season. Variation in isotopic niches between genotypes revealed that heterozygotes were moderately more enriched in δ^13^C of hair grown during fall foraging compared with black‐coated homozygotes

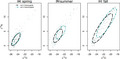

## 1 INTRODUCTION

Niche partitioning among and within species is a central process underlying biodiversity. An historical assumption underlying most research on niche variation is that niches are properties of populations or species (Chase & Leibold, 2003; Hutchinson, 1957); however, diversity has increasingly been shown to be additionally driven by niche partitioning among age‐classes (Cox & Davis Rabosky, 2013), sexes (Phillips et al., 2004), and individuals (Araújo et al. 2011; Newsome et al. 2009). Moreover, early theoretical and empirical work revealed the potential for niche differentiation between genotypes and/or phenotypes (“multiniche polymorphisms”) to maintain intraspecific diversity (Ford, 1975; Reimchen, 1979). Specifically, it has been proposed that the diversity of discrete phenotypes (via genotypes) can be maintained by occupying distinct niches across different axes (i.e., habitat, diet) whereby the fitness of each morph is paired to the appropriate niche (Levene, 1953; Van Valen, 1965). Additional empirical case studies of niche–phenotype associations may offer insight into the generalizability of this proposed mechanism across taxa and ecosystems.

Intraspecific body color variation is common in vertebrates, and increasing evidence has shown niche divergence among color morphs. Color polymorphisms occur across taxa including mammals (Majerus & Mundy, 2003), birds (Galeotti et al. 2003), and reptiles (Rosenblum et al. 2004). In birds, a recent literature review demonstrated that in 13 out of 16 polymorphic bird species, different color morphs of the same population foraged or bred in different habitats (Roulin, 2004). Among terrestrial mammals, coat color is also often associated with habitat, suggesting a prominent role for camouflage (Caro, 2005; Suzuki, 2013). For example, rock pocket mice (*Chaetodipus intermedius*) have dark and light coat color morphs, with light morphs selecting for habitat with lighter substrates and dark morphs selecting for dark substrates (Hoekstra et al. 2004; Nachman et al. 2003). An individual's color can also dictate the food resources available to them and their dietary niche. For example, reddish‐brown barn owls (*Tyto alba*) foraged on field voles (*Microtus arvalis*) more often, whereas conspecifics with lighter plumage consumed more wood mice (*Apodemus* spp.; Roulin, 2004). Given their conspicuous nature and relationship to niche divergence, color polymorphisms provide an exceptional opportunity to study the maintenance of intraspecific diversity.

Coastal British Columbia (BC) hosts one of the most striking coat color polymorphisms known in mammals: a rare white‐coated morph of the American black bear (*Ursus americanus kermodei;* Ritland et al. 2001). Referred to commonly as a “Spirit bear,” genetic research has identified its unique coat color to be controlled by a recessive mutation at the melanocortin 1 receptor (*mc1r*) gene (Ritland et al. 2001). Heterozygotes and dominant homozygote bears have black coats and are visually indistinguishable from each other (Ritland et al. 2001). Spirit bears coexist with black‐coated black bears and have a limited distribution, primarily on several islands and the nearby mainland on the central coast of BC (Marshall & Ritland, 2002; Ritland et al. 2001). In this region, frequencies of the Spirit bear phenotype have been estimated as high as 43% (Gribbell Island), but their probability of presence drops to near zero within ~3 km from this concentrated area (Marshall & Ritland, 2002; Ritland et al. 2001; Service et al., 2020). Population estimates throughout their distribution vary from ~50 to 500 individuals (Darimont, unpublished data; Blood, 1997; McCrory et al. 2001; Sachs, 2010).

The evolutionary and ecological context that supports this unusual polymorphism, and specifically the interplay between neutral and selective forces, is yet to be resolved. The Kitasoo/Xai'xais First Nation's oral history from the area documents the presence of white bears at similar frequencies to present day since the Wisconsin glaciation (Carter, 1966), suggesting that the polymorphism is stable. Hedrick and Ritland (2011) assessed the theoretical impact of selection, genetic drift, gene flow, and positive assortative mating on coastal black bear populations in the region to consider the conditions that could support the maintenance and restricted distribution of the white coat polymorphism. These models posit the importance of genetic drift in the establishment of the white allele, but suggested that a selective advantage was critical for counteracting gene flow from homozygous populations without the white allele (Hedrick & Ritland, 2011).

Recent evidence suggests the importance of foraging behavior in the selective landscape. Klinka and Reimchen (2009) showed that relative to the black morph (*n* = 37), white individuals in their study (*n* = 4) had increased salmon capture rates during daylight, part of which could be attributed to reduced evasiveness of salmon to the white morph, as inferred from in‐stream experimental observations using a simulated predator. Subsequent dual stable isotope (δ^13^C and δ^15^N) analysis demonstrated enriched signatures in the white morph relative to the black morph, which may be indicative of increased salmon consumption (Reimchen & Klinka, 2017). The extent of this enrichment was found to be proportional to the frequency of the white morph among islands (Reimchen & Klinka, 2017). Whereas increased salmon consumption is likely, enriched signatures could also indicate foraging at higher trophic levels (Ben‐David & Flaherty, 2012) or nutritional stress (Cherel et al. 2005; Hobson et al. 1993). Given these results, the authors have suggested that ecological segregation has promoted the persistence of this polymorphism through a multiniche mechanism (Klinka & Reimchen, 2009; Reimchen & Klinka, 2017). However, how selection might act on black‐coated heterozygotes and how such selection may contribute to the maintenance of this polymorphism has not yet been confronted with detailed data. Previous research on the frequency of heterozygotes at a landscape scale had suggested that selection may be acting upon heterozygotes (Ritland et al. 2001), though this pattern was not detected in a recent analysis (Service et al., 2020). Differential selection among genotypes that share the same phenotype of coat color has been demonstrated in wolves (*Canis lupus*; Coulson et al. 2011), but the potential role of differences in fitness among genotypes with the same coat color is uncertain in this system.

Stable isotope analysis provides a useful tool to account for and characterize the multidimensional foraging niche space between phenotypes and genotypes in this coastal bear system, and likely many others. Specifically, δ^13^C and δ^15^N can differentiate foraging niche by terrestrial versus marine diet (elevated δ^13^C and δ^15^N in marine foods) and trophic level (elevated δ^15^N with each trophic step) (Ben‐David & Flaherty, 2012; Lafferty et al. 2015). For coastal bears, the landscape of their foraging niche is thought to be dominated by terrestrial plant matter (low δ^13^C and δ^15^N relative to other foods available to bears in this system), intertidal resources (high δ^13^C and lower δ^15^N), and spawning Pacific salmon (*Oncorhynchus* spp., as indicated by elevated δ^13^C and δ^15^N; Hilderbrand et al. 1999, Adams et al. 2017, Service et al. 2018). These dual isotopes are often used in coastal bear studies for their ability to clearly differentiate among food items with differing fitness implications. For example, increased salmon consumption has been demonstrated to increase litter size, and body size, as well as improve body condition (Hilderbrand et al. 1999), growth of cubs, and denning survival (Ben‐David et al., 2004). These benefits in turn have been observed to scale the population level; across multiple populations in North America, salmon consumption correlates with higher densities in an approximately 1:1 relationship (i.e., ~25% increase in the populations salmon consumption is correlated with an ~25% increase in population density; Hilderbrand et al. 1999). Importantly, although enriched isotopic values can arise from other processes (seasonal metabolic changes, nutritional stress, trophic level), captive feeding trials of bears have demonstrated that an increase in salmon consumption is reliably correlated to increased δ^13^C and δ^15^N (Hilderbrand et al. 1996). As a result, even modest enrichment of δ^13^C and δ^15^N, a consistently demonstrated signal of increased salmon consumption, may translate to fitness benefits.

Although the description of the classical niche concept describes a “n‐dimensional hypervolume” (Hutchinson, 1957), many ecological investigations into intraspecific niche variation have examined only one axis (e.g., prey size, δ^13^C, δ^15^N) at a time. Such univariate approaches have recently been shown to affect estimates of niche overlap (Friedemann et al. 2016; Ingram et al. 2018; Pulla et al. 2017). In response, a recently proposed analytical framework addresses the discrepancy between the commonly estimated univariate niche axis and the multivariate nature of conceptualized niche space by employing multivariate mixed‐effects models (Ingram et al. 2018).

We use this multivariate stable isotope approach in a novel application to characterize and compare the foraging niche between phenotypes (white versus black coat) and genotypes (black‐coated heterozygote versus black‐coated homozygote) of coastal black bears. We draw inference at both an annual and seasonal temporal scale to examine the hypothesis that a multiniche model underlies this polymorphism. Specifically, we predicted a more marine‐based foraging niche in Spirit bears compared with black‐coated black bears. Given conflicting evidence within this system (an observed deficiency in heterozygotes, but a seemingly balanced polymorphism, which could be maintained in part by heterozygote advantage), we additionally predicted that black‐coated heterozygotes and black‐coated homozygotes may diverge in foraging niche, but did not predict direction of such divergence.

## 2 METHODS AND MATERIALS

### 2.1 Study area

**FIGURE 1:**
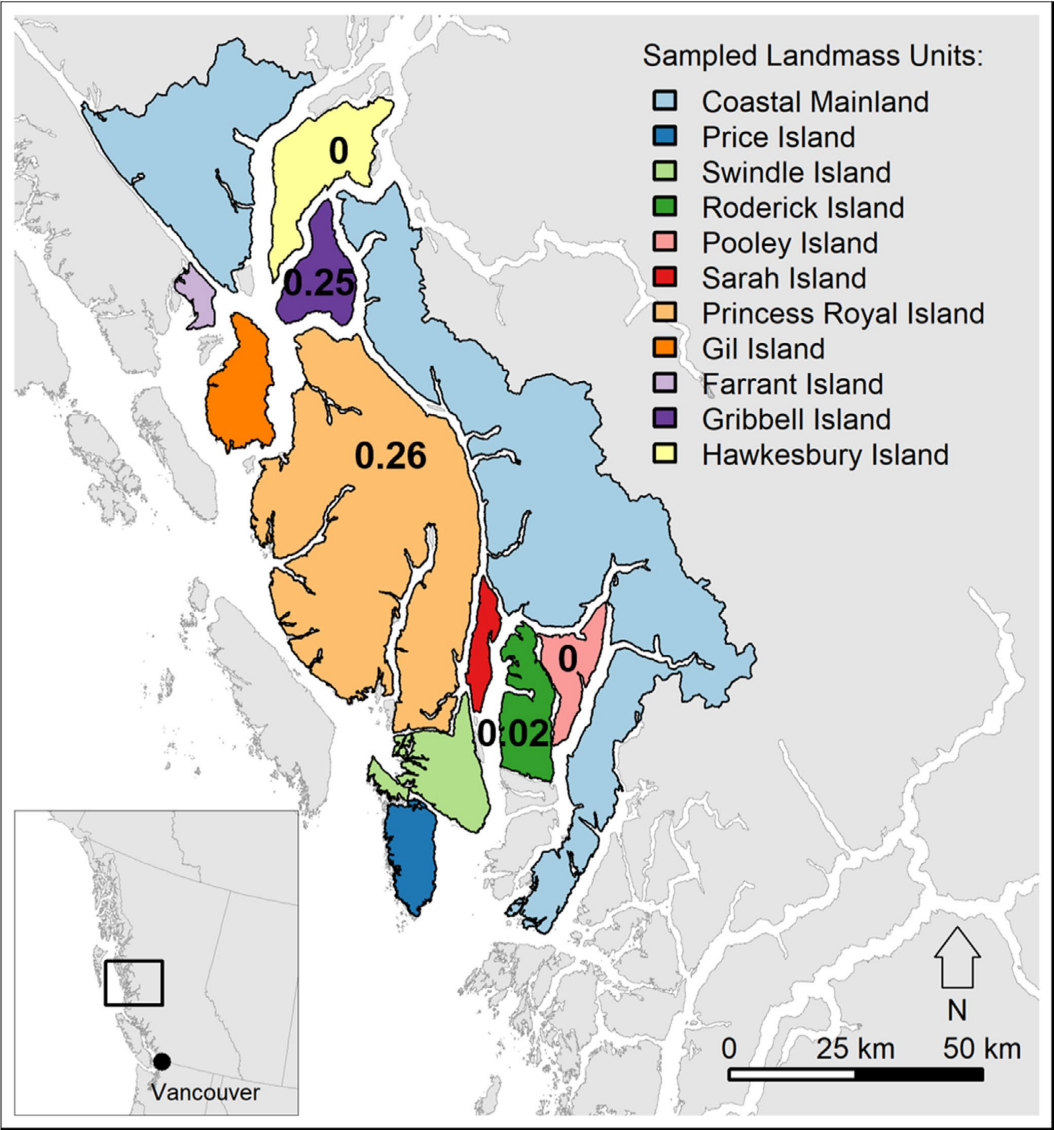
Study area as defined by the extent of sampled “landmass units” in coastal British Columbia, Canada. Bolded numbers overlaid on select landmass units indicate (where available) the most recent *mc1r* G allele frequency data (Service et al., 2020)

Our remote study area—commonly referred to as the “Great Bear Rainforest”—consists of coastal islands, and adjacent mainland regions of temperate rainforest previously reported to host Spirit bear alleles on the central coast of British Columbia, Canada (Marshall & Ritland, 2002; Figure 1). Black bears are present throughout the entire region, whereas grizzly bears (*U. arctos horribilis*) primarily occur in mainland watersheds (Service et al. 2014). This region consists of the contemporary and traditional territories of the Gitga'at and Kitasoo/Xai'xais First Nations, among others, and their communities of Hartley Bay and Klemtu, respectively.

Throughout the study area, black bears have access to spawning salmon (predominately pink [*O. gorbuscha*], chum [*O. keta*], coho [*O. kisutch*], and sockeye [*O. tshawytscha*]) in the fall (August–November; peak salmon availability September–October), and plant‐based foods (e.g., berries, roots, emergent vegetation, sedges), and intertidal resources (e.g., mussels, crabs, and other shoreline invertebrates) throughout the year (Adams et al. 2017). Contributions of terrestrial meat to diet are negligible (Adams et al. 2017). Bears in the region target salmon when they return to spawn in fall months because of their high lipid and protein content, offering a valuable food source prior to winter sleep (Ben‐David et al. 2004; Hilderbrand et al. 1999). The nutritional benefits from salmon have also been shown to have population‐level implications (i.e., higher densities) for coastal bear populations (Hilderbrand et al. 1999).

### 2.2 Field sampling

Owing to sampling limitations related to this very rare polymorphism, we used two spatially complementary, but temporally exclusive, datasets to address our objectives. We refer to them as the *phenotype* and *genotype* datasets. The phenotype dataset compares potential niche divergence between white‐ and black‐coated color morphs (i.e., GG and pooled AG and AA genotypes, respectively). The genotype dataset distinguishes between the black‐coated heterozygote and black‐coated homozygote individuals (i.e., AG and AA genotypes).

#### 2.2.1 Phenotype dataset

The phenotype dataset consists of hair samples of individual bears collected during fall (September, October) between 1997 and 2000 by K. Ritland et al. (Marshall & Ritland, 2002; Ritland et al. 2001; *n* = 35; 17 black coat; 18 white coat). Samples were obtained from passive, scent‐lured corral hair snags located on bear trails near salmon‐bearing rivers (*n* = 11 hair snags with samples that contribute to this analysis; full details of Ritland et al.’s approach in Marshall & Ritland, 2002; Ritland et al. 2001). Although we anticipate that the placement of these hair snags near salmon rivers may have resulted in sampling bears that consume more salmon than the population as a whole, we have no reason to believe that this site placement would lead toward any directional bias in salmon consumption between color morphs. These samples were subsequently provided to D. Klinka and T. Reimchen by K. Ritland (Klinka, 2004; Reimchen & Klinka, 2017), and we used our novel multivariate approach to re‐examine the δ^13^C and δ^15^N data from these samples.

#### 2.2.2 Genotype dataset

The genotype dataset contains hair samples from 143 black‐coated individuals (30 AG heterozygote and 113 AA homozygote). Despite six seasons of sampling over ~22,000 km^2^, which identified 148 genotyped individuals with sufficient material for segmented isotopic analysis, only five were white‐coated homozygotic genotypes. Consequently, they could not be considered in this analysis.

Using approximately evenly spaced (~1 per 50 km^2^) noninvasive hair snagging sites (mean *n* = 103; range *n* = 77–128 per year) baited with a nonreward scent lure bait (Woods et al. 1999, details in Bryan et al. 2013; Bryan et al. 2014, Adams et al. 2017, Service et al. 2018), we collected bear hair samples for the genotype dataset every ten to fourteen days during late spring (May and June). Sites consisted of a focal pile of vegetation that was baited with a liquid fish scent lure and then encircled by a 50‐cm high barbed wire corral. Trap spacing (~7 km apart) was determined to accommodate the broader research program's objectives of monitoring coastal grizzly and black bears (of which this study is one component). We collected hair in the southern half of the study area from 2012 through 2017 (*n* = 77 sites), and the northern half from 2015 to 2017 (*n* = 51 sites), and combined these regions for analysis. Sampling days (*n* ≈ 30–40 per site per year) were consistent across years. Through boat and helicopter access, we sampled across a range of elevations (0–574 m), and in salmon‐bearing and non‐salmon‐bearing watersheds. These hair samples were collected via research partnerships with the Gitga'at and Kitasoo Xai'xais First Nations.

Our protocol was approved by the Stewardship Departments of the Kitasoo/Xai'xais and Gitga'at First Nations. Sampling in Parks occurred under BC Parks Use Permit 108648. Research was approved by the University of Victoria's Animal Care protocol 2016‐020 and followed the Canadian Council for Animal Care's requirements concerning animal care and wildlife (Sikes & Gannon, 2011).

#### 2.2.3 Dataset comparisons

Due to important differences between these datasets, we were not able to directly compare them. First, the shedding phase of the annual molt occurs during our collection period in late spring (~May), and as such, we assumed the spring‐collected hair (i.e., fully grown strands) in the genotype dataset reflects foraging in the previous year during the hair growth stage which lasts from ~May to late fall–winter sleep (Hilderbrand et al. 1996; Jones et al. 2006; Schwartz et al. 2003). In contrast, the fall hair in the phenotype dataset records only the period of growth up to the point during fall growth when the hair was sampled (up to ~8 weeks before predicted winter sleep). Given these differences, the base, mid‐, and tip segments of both these datasets correspond to different time periods of foraging (~fall, summer, and spring, respectively), but do not correspond with each other (each segment representing ~1.5 months in the phenotype dataset and ~2.3 months in the genotype dataset). Additionally, as the phenotype dataset consists of samples exclusively collected near salmon‐bearing rivers, we would expect this dataset to be biased toward salmon isotope signatures compared with the genotype dataset, which sampled across salmon and non‐salmon watersheds and a gradient of elevations. Owing to these fundamental differences, we separately examined hypotheses related to niche divergence between phenotypes and subsequently among genotypes, using near‐identical methods applied to these parallel datasets.

### 2.3 Genetic analyses

For the phenotype dataset, the original samples contained information on individual identity, as determined by eight microsatellite loci, and color morph (Marshall & Ritland, 2002). Sex data were not available.

For the samples contributing to the genotype dataset, we contracted a commercial laboratory, Wildlife Genetics International, to conduct genetic analyses (Wildlife Genetics International, Nelson, BC, Canada). Information from seven microsatellite loci plus an amelogenin locus sex marker revealed species (black vs. grizzly bear), sex, and individual identity. Genotypes at the *mc1r* locus were characterized as GG (recessive homozygote, white coat phenotype); AG (heterozygote, black coat phenotype); or AA (dominant homozygote, black coat phenotype) (Service et al., 2020).

### 2.4 Stable isotope laboratory analysis

We obtained the stable isotope values directly for the phenotype dataset from T. Reimchen (Klinka, 2004; Reimchen & Klinka, 2017), though the stable isotope laboratory analysis process was nearly identical for the genotype and phenotype datasets. For each sample for which polarity could be identified (i.e., presence of a follicle), the hair was cut into three equal length segments that were processed and analyzed separately (Klinka, 2004). To remove oils and surficial debris, segmented samples were washed and rinsed with a 2:1 mixture of chloroform: ethanol for the phenotype dataset and a 2:1 mixture of chloroform: methanol for the genotype dataset. For the phenotype dataset, after the samples were dried at 60°C for at least 48 hr, the hair was ground and ~ 1 mg subsampled into tin capsules. For the genotype dataset, samples were dried at room temperature for at least 48 hr, and then, each section was cut into small pieces; the hair sections were then homogenized through thorough mixing, and ~1 mg was subsampled into tin capsules. The samples from both datasets were subsequently analyzed using continuous‐flow isotope ratio mass spectrometry at the University of Saskatchewan (University of Saskatchewan, Canada; Darimont & Paquet, 2002; Reimchen & Klinka, 2017). Specifically, samples were analyzed using a Europa Scientific ANCA‐NT gas–solid–liquid preparation module coupled to a Europa Scientific Tracer 20–20 mass spectrometer in a sequence of ten unknowns, followed by two albumin standards. Analytical error was estimated to be ±0.05 ‰ (*SD*: 0.04) for carbon, and ±0.04‰ (*SD* 0.05) for nitrogen.

All isotope ratios are expressed as *δ* values, which report parts per mil (‰), according to the equation:

$$\delta X=(\left(R_{\text{sample}}/R_{\text{standard}}\right)‐1)$$

where *X* represents ^13^C or ^15^N, and *R* represents the ratio of heavy to light isotopes. Vienna‐Pee Dee Belemnite limestone (V‐PDB) and atmospheric N_2_ are the standard for carbon and nitrogen, respectively.

### 2.5 Statistical analysis of isotopic data

We applied the Bayesian multivariate linear mixed‐effects models, with δ^13^C and δ^15^N together comprising the response variable, to draw inference from data on potential foraging niche divergence between black bear phenotypes (black and white) and between homozygote black and heterozygous genotypes. Employing Markov chain Monte Carlo (MCMC) estimation in the package *MCMCglmm* (Hadfield, 2010), we ran 250,000 iterations for each model with a thinning interval of 50 and discarded the first 10,000 iterations as a burn‐in. Individual ID was included as a random effect in all models to account for repeated measures across seasons. We confirmed suitable mixing and convergence of all models by applying Gelman–Rubin diagnostic tests after running multiple chains with staggered starting points (Brooks & Gelman, 1998). All analyses were conducted in R Studio version 3.4.3 (R Core Team, 2018).

Variance–covariance matrices from multivariate linear mixed‐effects models were visualized as standard ellipses in δ^13^C and δ^15^N isotopic space to assess potential niche variation as described by metrics of area and overlap (Ingram et al. 2018; Jackson et al. 2011). In line with previous bivariate isotopic foraging studies, we compared phenotypes and genotypes using “core” foraging niche areas represented by standard ellipses, which are expected to contain approximately 40% of bivariate isotope data regardless of sample size (Jackson et al. 2011; Lafferty et al. 2015). Additionally, we compared model parameters (i.e., coefficient estimates) directly to assess whether the 95% credible intervals for parameters overlapped zero. We analyzed variation in isotopic data using annual and seasonal measures of resource use (δ^13^C and δ^15^N).

#### 2.5.1 Annual dietary niche variation

We analyzed annual niche variation with a Bayesian MLMM*,* with individual ID included as a random effect. As all hair was segmented, each individual was represented by three samples corresponding to three time periods. The variances and covariances among individual mean isotope values based on the random effect of ID were interpreted as the multivariate between‐individual component (BIC) of the population niche. The residual variances and covariances were taken to represent the within‐individual component (WIC) of the population niche, and the BIC and WIC matrices were added together to estimate the population multivariate total niche width (TNW). The TNW matrix for a population was represented graphically using standard ellipses, and the size of the TNW matrix was calculated as the sum of its eigenvalues (Ingram et al. 2018). Area of the standard ellipse and overlap between phenotypes and genotypes was calculated in the R package *spatstat:utils* (version 1.13‐0). Ninety‐five percent credible intervals of overlap and area estimates were determined by subsampling every 100th iteration of the Markov chain Monte Carlo model. As TNW ellipse shape and size could vary within phenotypes and genotypes, overlap percentages could differ when calculated for Ellipse A (i.e., white phenotype or homozygote genotype) in Ellipse B (i.e., black phenotype or heterozygote genotype) versus Ellipse B in Ellipse A. Accordingly, we report two asymmetric overlap metrics for each phenotype/genotype comparison at the annual temporal scale. Additionally, we examined model parameters estimated with the phenotype and genotype analyses to assess the magnitude and direction of the effects of the “genotype” and “phenotype” predictors on both isotope values. Accordingly, the final annual model form from which we draw inference included both isotopes as response variables, a random effect (varying by both slope and intercept) for individual, and a fixed effect of either genotype or phenotype.

#### 2.5.2 Seasonal dietary niche variation

Seasonal dietary niche variation between phenotypes and genotypes was assessed by building more complex MLMMs that included random effects (varying by slope and intercept) of individual ID and landmass (location of detection summarized by island name or “coastal mainland”; Figure 1; *n* = 10 [phenotype dataset]; *n* = 7 [genotype dataset]). Landmass was included to account for features of the environment that vary across space and are known to influence foraging niche, but that were not directly related to our simplified hypotheses, such as competitive environment (Service et al. 2014), *mc1r* allele frequency (Reimchen & Klinka, 2017), and salmon availability (Service et al. 2018). To account for known sexual dimorphism in black bear foraging (Adams et al. 2017), sex was also included as a fixed effect in the genotype models. As sex was not a parameter of interest, we fixed the sex parameter's contributions to the MLMM at its mean value to visualize the TNW areas of each genotype (no sex data were available in the phenotype dataset; sex‐specific genotype TNW shown in SI Appendix S1). As above, the Bayesian ellipse estimation by season was used to calculate area and overlap of core isotopic niches between genotypes and between phenotypes. To avoid overparameterization, we allowed the TNW area to vary between seasons, but not within phenotypes and genotypes. As such, we report only one overlap value for each season. Finally, we assessed mean model parameter estimates and credible intervals to determine the magnitude and direction of effects of the phenotype or genotype predictors.

## 3 RESULTS

### 3.1 Stable isotope values

Isotopic values across individuals and between seasons were variable in both the phenotype and genotype datasets: (a) phenotype dataset: δ^15^N: mean (*SD*) = 4.36‰ (3.33) and δ^13^C: mean (*SD*) = −22.84‰ (1.80), and (b) genotype dataset: δ^15^N: mean (*SD*) = 5.23‰ (4.01) and δ^13^C: mean (*SD*) = −22.88‰ (2.24).

### 3.2 Annual dietary niche variation between phenotypes and among genotypes

**FIGURE 2:**
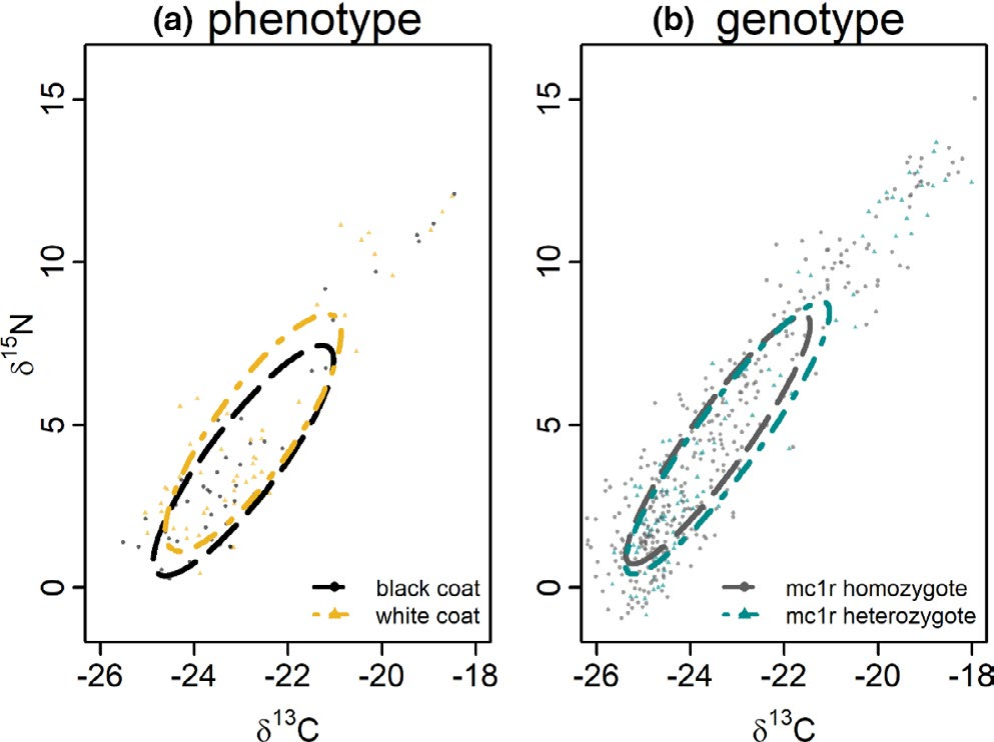
Annual δ^13^C and δ^15^N isotopic foraging niche variation in coastal black bears (*Ursus americanus kermodei*) between a) coat color phenotype (*n* = 35; 17 black coat; and 18 white coat) and b) *mc1r* dominant homozygote and heterozygote genotypes (*n* = 143; 30 AG heterozygote and 113 AA homozygote). Ellipses represent core total niche width (TNW) as inferred by a multivariate repeated‐measures Bayesian linear mixed‐effect models. Points are raw data observations of tip, mid‐, and base hair segments of each detected individual

Ellipses representing annual core TNW did not vary substantially in area between phenotypes (black coat: mean 9.9, 95% CI: 7.5–13.9; white coat: mean 11.0, 95% CI: 8.4–15.5). Overlap in TNW ellipses between phenotypes, as measured as a percentage of a total area, was 86% (95% CI: 47%–99%) between black‐ and white‐coated individuals and 76% (95% CI: 41%–94%) between white‐ and black‐coated individuals (Figure 2a). Coefficient parameter values from the core MLMM revealed that phenotype had no influence on isotopic means (Table 1).

**TABLE 1:** Parameter coefficient estimates and associated 95% credible intervals for the Bayesian multivariate linear mixed models that relate δ^13^C and δ^15^N values of coastal black bear (*Ursus americanus kermodei*) hair samples to their phenotype and genotype

Model	Effect	Mean estimate	U – 95%	L – 95%	Effective *n*	pMCMC
Annual phenotype	**Intercept**	−23.0	−23.6	−22.4	4,800	**~0.00**
	**δ^15^N**	26.8	26.0	27.5	4,800	**~0.00**
	Phenotype (white coat)	0.2	−0.7	1.0	4,575	0.65
	δ^15^N *phenotype (white coat)	0.7	−0.4	1.8	4,800	0.22
Annual genotype	**Intercept**	−23.1	−23.6	−22.6	4,800	**~0.00**
	**δ^15^N**	28.0	27.5	28.6	4,800	**~0.00**
	Genotype (AG)	0.7	−0.4	1.7	4,439	0.21
	δ^15^N *genotype (AG)	0.2	−0.9	1.3	4,800	0.67
Seasonal phenotype	**Intercept**	−21.7	−23.1	−20.4	4,800	**~0.00**
	**δ^15^N**	28.1	26.1	30.2	4,800	**~0.00**
	Phenotype (white coat)	0.2	−1.2	1.5	4,929	0.77
	**seg (mid)**	−1.8	−2.9	−0.8	4,586	**~0.00**
	**seg (tip)**	−2.6	−3.7	−1.5	4,800	**~0.00**
	δ^15^N *phenotype(white coat)	1.2	−0.2	2.8	4,800	0.09
	δ^15^N *seg (mid)	−1.7	−2.9	−0.4	4,800	0.09
	δ^15^N *seg (tip)	−2.0	−3.2	−0.7	4,800	**0.01**
	Phenotype(white coat)*seg (mid)	−0.20	−1.7	1.3	4,920	0.78
	Phenotype(white coat)*seg (tip)	0.3	−1.2	1.7	4,981	0.72
	δ^15^N *phenotype(white coat)*seg (mid)	−0.8	−2.6	0.9	5,022	0.38
	δ^15^N *phenotype(white coat)*seg (tip)	−0.8	−2.6	1.0	4,800	0.37
Seasonal genotype	**Intercept**	−22.6	−23.6	−21.1	4,572	**~0.00**
	**δ^15^N**	27.8	26.9	28.9	4,800	**~0.00**
	**Sex (male)**	0.5	0.2	0.9	4,800	**0.01**
	Genotype (AG)	1.1	−0.2	2.4	4,800	0.09
	**seg (mid)**	−1.5	−2.1	−0.9	4,812	**~0.00**
	**seg (tip)**	−2.3	−2.9	−1.7	4,800	**~0.00**
	δ^15^N *sex (male)	0.7	0.2	1.1	4,800	**~0.00**
	δ^15^N *genotype (AG)	0.8	−0.4	2.0	4,800	0.19
	δ^15^N *seg (mid)	−1.1	−1.1	−0.5	4,587	**~0.00**
	δ^15^N *seg (tip)	−1.6	−2.2	−1.0	4,800	**~0.00**
	Genotype (AG)*seg (mid)	−0.3	−1.4	0.9	4,800	0.62
	Genotype (AG)*seg (tip)	−0.9	−2.1	0.2	4,800	0.11
	δ^15^N *genotype (AG)*seg (mid)	−0.4	−1.5	0.6	4,800	0.41
	δ^15^N *genotype (AG)*seg (tip)	−0.9	−1.9	0.2	4,800	0.10

Note

Seasonal models include interactions with hair segment as a proxy for foraging season, with tip, mid, and base representing spring, summer, and fall, respectively. All models include individual ID as a random effect to account for the repeated‐measures structure that results from each bear contributing three hair segments. Both seasonal models include landmass (island name or “coastal mainland”) as a random effect and the genotype model additionally includes sex as a fixed effect. We used δ^13^C values, black phenotypes, the female sex, AA genotypes, and base hair segments as reference conditions for all models for which those terms apply. Parameters with coefficient estimates that did not overlap zero are bolded.

We did not detect a difference in isotopic foraging niche area in annual comparisons between genotypes with an estimated mean TNW area for homozygotes of 11.00 (95% CI: 7.9–10.1) compared with 10.7 for heterozygotes (95% CI: 8.6–13.7) (Figure 2b). Similar to phenotypic comparisons, niche overlap was high among genotypes. Homozygote/heterozygote overlap was 87% (95% CI: 66%–100%), whereas heterozygote/homozygote overlap was 71% (95% CI: 54%–90%). Additionally, the core MLMM did not detect an influence of genotype on isotopic niche at the annual temporal scale (Table 1).

### 3.3 Seasonal comparisons

In both the phenotype and the genotype datasets, the base (fall) hair segment was the most enriched in ^15^N and ^13^C. The tip (spring) segment was consistently the most depleted in both isotopes, and the mid (summer)‐segment was indistinguishable from the tip (Table 1).

We did not detect a difference in the core TNW area across seasons in the phenotype dataset (fall mean: 6.4 (95% CI: 2.4–17.7); summer mean: 3.4 (95% CI: 2.0–5.5); spring mean: 3.5 (95% CI: 1.9–5.7)). In contrast, seasonal niche TNW area diverged in the genotype dataset; the core niche area was the largest in the fall (genotype mean: 15.0 (95% CI: 6.9–27.1)), with a smaller and similarly sized TNW area for both summer (phenotype mean: 2.6 (95% CI: 1.3–4.6)) and spring segments (genotype mean: 3.3 (95% CI: 1.5–5.6)).

**FIGURE 3:**
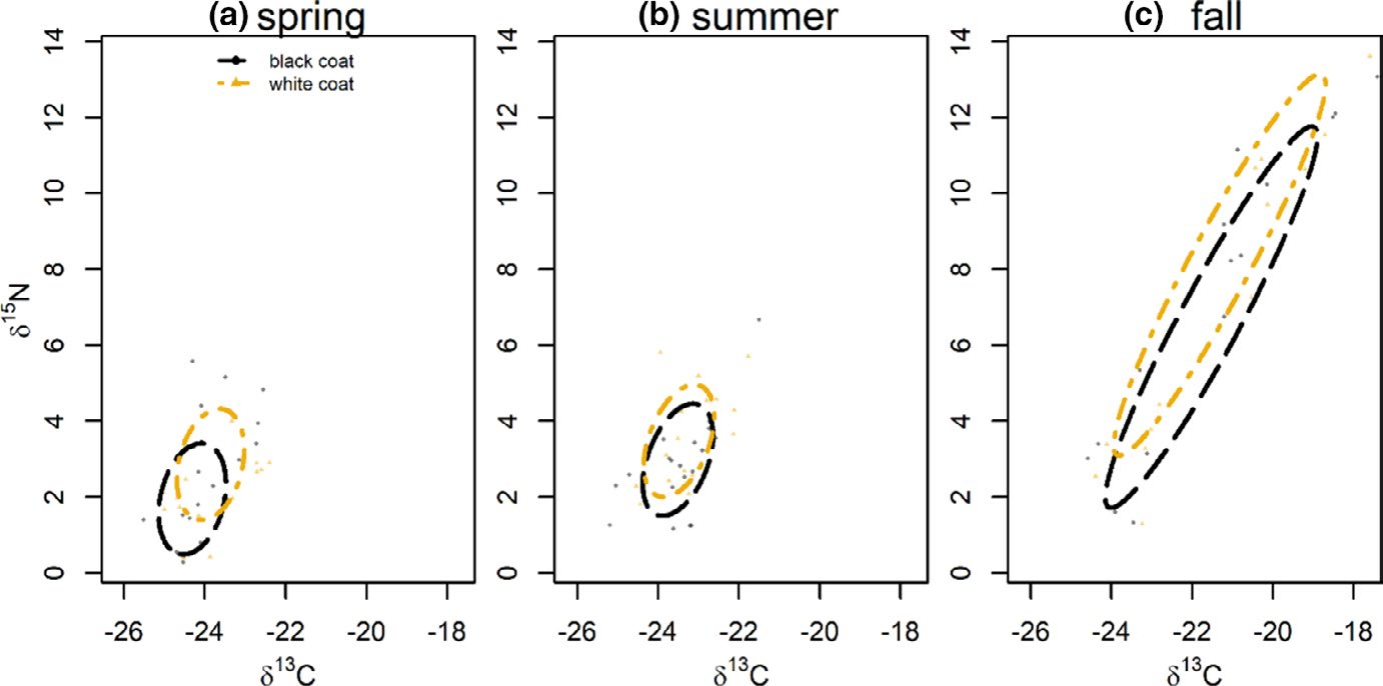
Seasonal isotopic foraging niche variation (δ^13^C and δ^15^N) in coastal black bears (*Ursus americanus kermodei*) between coat color phenotypes (*n* = 35; 17 black coat; and 18 white coat) during a) spring (tip hair segment), b) summer (midsegment), and c) fall (base segment). Ellipses represent core niche area as inferred by a multivariate repeated‐measures Bayesian linear mixed‐effect models. Semitransparent black and yellow points are raw data observations of tip, mid‐, and base hair segments of each detected individual

Though results were not statistically significant at an alpha value of 0.05, the white phenotype had a slight trend toward enrichment in ^15^N during the fall season (pMCMC: 0.09; Table 1; Figure 3). However, there was no directional pattern in ^13^C differences between phenotypes across all seasons. Additionally, niche overlap varied across seasons (spring: 55% (95% CI: 9–87); summer: 75% (95% CI: 25–92); fall 63% (95% CI: 0–77)).

Our analysis of the genotype dataset revealed that heterozygotes were moderately more enriched in ^13^C in the base segment of hair that represents fall foraging (pMCMC: 0.09; Table 1), though these results were not statistically significant at an alpha value of 0.05. Additionally, though 95% credible intervals overlapped zero indicating lack of statistical significance, tip hair segments of homozygotes were modestly enriched in both ^13^C and ^15^N compared with heterozygotes (pMCMC ^13^C tip: 0.11; pMCMC ^15^N tip: 0.10; Table 1). No directional pattern was observed in the remaining seasons across both isotopes and mean overlap varied across seasons (spring: 58% (95% CI: 4%–94%); summer: 32% (95% CI: 0%–83%); fall: 64% (95% CI: 26%–93%)).

## 4 DISCUSSION

**FIGURE 4:**
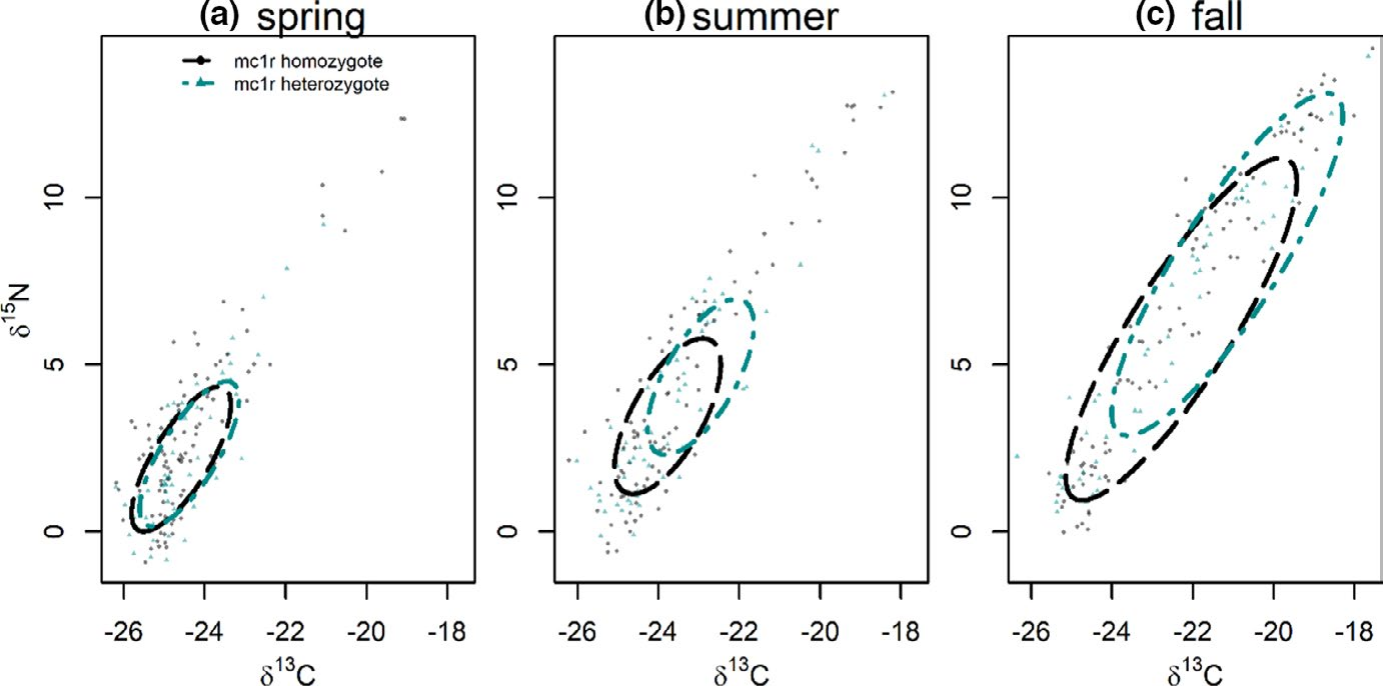
Seasonal isotopic foraging niche variation (δ^13^C and δ^15^N) in coastal black bears (*Ursus americanus kermodei*) between dominant homozygote and heterozygote *mc1r* genotypes (*n* = 143; 30 AG heterozygote and 113 AA homozygote) during (a) spring (tip hair segment), (b) summer (midsegment), and (c) fall (base segment). Ellipses represent core niche area as inferred by multivariate repeated‐measures Bayesian linear mixed‐effect models. Semitransparent black and teal points are raw data observations of tip, mid‐, and base hair segments of each detected individual

Our results suggest that both phenotypes and genotypes diverge modestly in foraging niche at a seasonal temporal scale (Figures 3 and 4). Although no MLMM parameters related to genotype and phenotype were statistically significant based on 95% credibility intervals, the directional pattern was consistent toward enrichment for heterozygote genotypes and white phenotypes (Table 1). Differentiation was most pronounced in the base hair segment, which represents fall foraging, suggesting the potential role of the arrival spawning salmon underlying this divergence.

Across phenotypes and genotypes, TNW area was largest in the fall and smaller in the spring and summer (Figures 3 and 4). This pattern suggests that black bears widen their niche breadth in response to the additional resource availability afforded by salmon, rather than exclusively prey switching to this lucrative resource (Hilderbrand et al. 1999). This result is consistent with recent studies across taxa, which demonstrate that populations of generalist consumers with access to a variety of prey types may be composed of individuals whose dietary niches are smaller subsets of the population's total niche width (Bearhop et al. 2004; Bolnick et al. 2007; Reimchen et al. 2019).

The small sample sizes and reliance on noninvasive (i.e., hair based) methods inherent with studying rare organisms pose several limitations to our work. Specifically, we are unable to make direct comparisons of the magnitude of niche differentiation across the phenotype and genotype datasets, given the difference in their temporal scope (spring to early/mid‐fall vs. spring to late fall–winter sleep) and period of hair collection (1990s vs. 2010s). Additionally, we acknowledge that our strength of inference and potential model complexity is limited by modest sample sizes. For example, we were not able to address annual variation in our analysis by including “year” in our models. Moreover, our reliance on comparisons among and between segmented hair samples depends on assumptions of consistent guard hair growth rate throughout the growing season as well as timing of hair growth onset and cessation (Jones et al. 2006). We recognize that several factors can influence these assumptions including nutritional status and seasonal changes in metabolic processes (Jacoby et al. 1999; Jones et al. 2006). However, our results from both datasets consistently indicated that the base (fall) hair segments were the most enriched in ^15^N and ^13^C, while the tip (spring) segments were most depleted. This directional pattern tracks our understanding of the seasonally changing isotopic landscape among available foods in this system (i.e., the arrival of salmon in the fall). These limitations highlight the importance of continued research and monitoring investment of this polymorphism of conservation concern.

Our observed pattern of a modest fall foraging divergence between phenotypes aligns with previous studies on Spirit bear foraging and provides context for the patterns we observed among genotypes. Reimchen and Klinka (2017) provided stable isotope‐based evidence of phenotypic niche divergence with white morphs on Gribbell Island (where previous genetic work suggested *mc1r* G allele frequency was highest) demonstrating elevated δ^15^N across all seasons but with a pronounced departure during fall foraging. This result suggested the role of a multiniche mechanism in supporting this polymorphism. Additionally, their previous observational and experimental work suggested that the observed niche divergence might be attributed to the reduced evasiveness of salmon when approached by white‐bodied predator models compared with black‐bodied models during daylight hours (Klinka & Reimchen, 2009). Given the evidence that salmon consumption enhances the fitness of coastal bears (Bryan et al. 2014; Hilderbrand et al. 1999), these combined results support the hypothesis that the white morph could have a selective advantage over black‐coated black bears during the fall. The convergence of our multivariate analysis results with previous univariate, observational, and experimental research (Klinka & Reimchen, 2009; Reimchen & Klinka, 2017) provides additional support for the role of a multiniche mechanism in maintaining this rare morph.

The modest foraging niche divergence among genotypes requires broader consideration than camouflage. Specifically, the mechanism of reduced salmon evasiveness proposed to underlie niche divergence between phenotypes does not apply between visually indistinguishable black‐coated heterozygotes and homozygotes (Klinka & Reimchen, 2009). Our data cannot address mechanisms that may explain the pattern of divergence, but we offer several hypotheses. Owing to the prolonged period of female‐cub association, cubs likely learn about foraging strategies and feeding sites from their mothers (Gilbert, 1999; Mazur & Seher, 2008). Accordingly, the modestly elevated marine isotopic signal we observe in heterozygotes might result from a learned behavior from those black heterozygotes raised by white‐coated mothers that specialized on marine diets (Reimchen & Klinka, 2017). Additionally, the proposed ecological and geographic segregation of color morphs, with black morphs closer to forests and white morphs suggested to occur on average closer to marine habitat and its resources (Reimchen & Klinka, 2017) could also contribute to this observed pattern. Specifically, given that black bear cubs generally overlap their mother's home range (Rogers, 1987), generations of heterozygote cubs from white mothers could be occupying home ranges at the marine interface. This explanation aligns with the recently reported discrepancy between a shoreline‐only sampling program that reported higher G allele frequencies (Ritland et al. 2001) compared with a more systematic sampling approach across all elevations (Service et al., 2020). Finally, divergence between black‐coated genotypes could be could also be driven by a process previously not identified in the genetic architecture of the polymorphism. For example, genotypes at *mc1r* could be related to traits associated with foraging behavior through pleiotropy or genetic linkage (Allendorf et al. 2016). Accordingly, future research would benefit from the use of modern genomic tools to provide a more comprehensive understanding of the potential ecological associations.

Our results provide novel insight into the mechanisms that may contribute to the maintenance of this rare polymorphism. Broadly, they reaffirm the potential role of a multiniche polymorphism in maintaining this rare morph. Our analysis provides new detail into the role heterozygotes may play in this maintenance. Although black morphs (pooled heterozygote and homozygote genotypes) were previously assumed to have reduced fitness compared with the white morph (Klinka & Reimchen, 2009; Reimchen & Klinka, 2017), the potential niche divergence between heterozygotes and black homozygotes genotypes was unexamined. If the elevated marine signatures of heterozygotes relate to fitness in the same way it is considered for white morphs, selective advantage may be highest for white morphs, followed by black heterozygotes, and finally black homozygotes (Table 1; Figure 4). This structure of selection pressure over certain times and in specific environments (Svardal et al. 2015) would further support the continued maintenance of the G allele.

Despite their potential selective advantage under certain conditions, the persistence of individuals carrying the G allele (white‐coated homozygotes and black‐coated heterozygotes) has been challenged by numerous historical and contemporary factors. For example, salmon populations in the region, which individuals carrying G alleles appear to have an advantage in accessing, have been substantially reduced from historic levels (Gresh et al. 2000). Additionally, Spirit bears were targeted by hunters before they were protected through regulation, which could have reduced the prevalence of G alleles in this landscape (McCrory, 2012). Moreover, the interspecific competitive environment has recently shifted with the range expansion of grizzly bears (*Ursus arctos*) over the past 20 years onto several islands with the highest frequency of the G allele (Service et al. 2014). This change to the ecological community could impact G‐carrying individuals through direct predation by larger bodied grizzly bears (Palomares & Caro, 1999). The insular environment these bears primarily inhabit presents additional challenges to the persistence of the G allele. Specifically, the isolation and restricted area of these island environments can only support small population sizes, a demographic factor that should not in theory support the long‐term persistence of this polymorphism in the absence of selection (Traill et al. 2007).

Deeper understanding of intraspecific niche variation can inform conservation strategies that protect phenotypic diversity. Relevant to coastal Spirit bear populations, individuals carrying the G allele (white dominant homozygotes and black‐coated homozygote) appear to diverge in foraging niche by occupying a more marine‐based diet. Accordingly, conservation action might maximize benefit to these bears by policy prescriptions that protect marine resources. Such management action would be particularly timely against a backdrop of reduced Pacific salmon returns (Price et al. 2017), industrial logging operations in the region's salmon‐bearing watersheds (McCrory, 2012), and realized potential marine contamination risks associated with industrial marine shipping accidents in the area (Heiltsuk Tribal Council, 2018). Against this context of cumulative environmental stressors, targeted conservation action that benefits niche diversity can play a significant role in maintaining biodiversity.

## CONFLICT OF INTEREST

The authors declare no conflict of interest.

## AUTHOR CONTRIBUTION

**Christina N. Service:** Conceptualization (equal); Formal analysis (lead); Investigation (lead); Methodology (equal); Project administration (equal); Writing‐original draft (lead); Writing‐review & editing (equal). **Travis Ingram:** Formal analysis (supporting); Methodology (equal); Resources (equal); Supervision (equal); Writing‐review & editing (equal). **Thomas E.** **Reimchen:** Conceptualization (equal); Investigation (equal); Resources (equal); Supervision (equal); Writing‐review & editing (equal). **Chris T. Darimont:** Conceptualization (equal); Funding acquisition (equal); Investigation (equal); Project administration (equal); Resources (lead); Supervision (lead); Writing‐original draft (equal); Writing‐review & editing (equal).

## Supporting information

Appendix S1

## Data Availability

Data are available from the Dryad Digital Repository at https://doi.org/10.5061/dryad.tdz08kpzn. Due to agreements with partnering Indigenous Nations, precise sampling locations are not shared.
