# Supplementary material for: Intrapopulation foraging niche variation between phenotypes and genotypes of Spirit bear populations
Source: Ecol Evol. 2021 Apr 13;11(10):5025–37. doi: 10.1002/ece3.7276 (PMC8131816; doi:10.1002/ece3.7276)
Supplement: Supplementary file 1 — Appendix S1 [file ECE3-11-5025-s001.pdf]

Supporting Information for ‘Intrapopulation foraging niche variation between phenotypes and genotypes of Spirit bear populations’

Authors: Christina N. Service, Travis Ingram, Thomas E. Reimchen, and Chris T. Darimont

***Corresponding author:** Christina Service, Kitasoo Xai’xais Stewardship Authority, Kitasoo/Xai’xais First Nation, 37 Raven Rd, PO Box 87, Klemtu, British Columbia, Canada, V0T 1L0. christina.service@gmail.com. 1.250.580.7302.

Appendix 1:

Supporting figures of TNW seasonal sex comparisons between mc1r genotypes of coastal black bears (*Ursus americanus kermodei*).

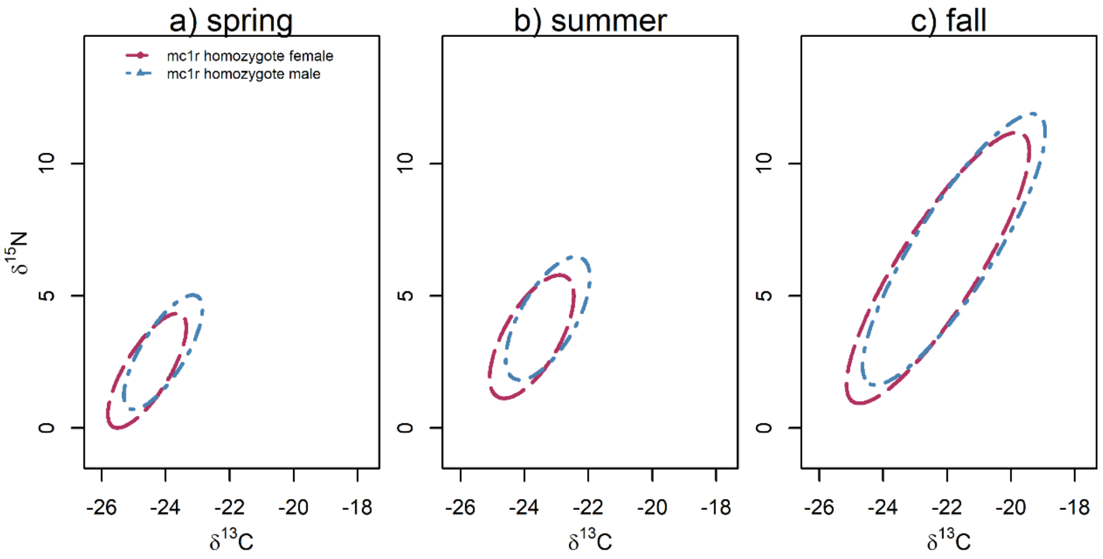

Figure S1. Seasonal isotopic foraging niche variation ($\delta^{13}C$ and $\delta^{15}N$) in coastal black bears (*Ursus americanus kermodei*) between male and female *mc1r* (AA) dominant genotypes (n = 21 female, n = 92 male) during a) spring (tip hair segment), b) summer (mid segment) and c) fall (base segment). Ellipses represent core niche area as inferred by multivariate repeated measures Bayesian linear mixed effect models.

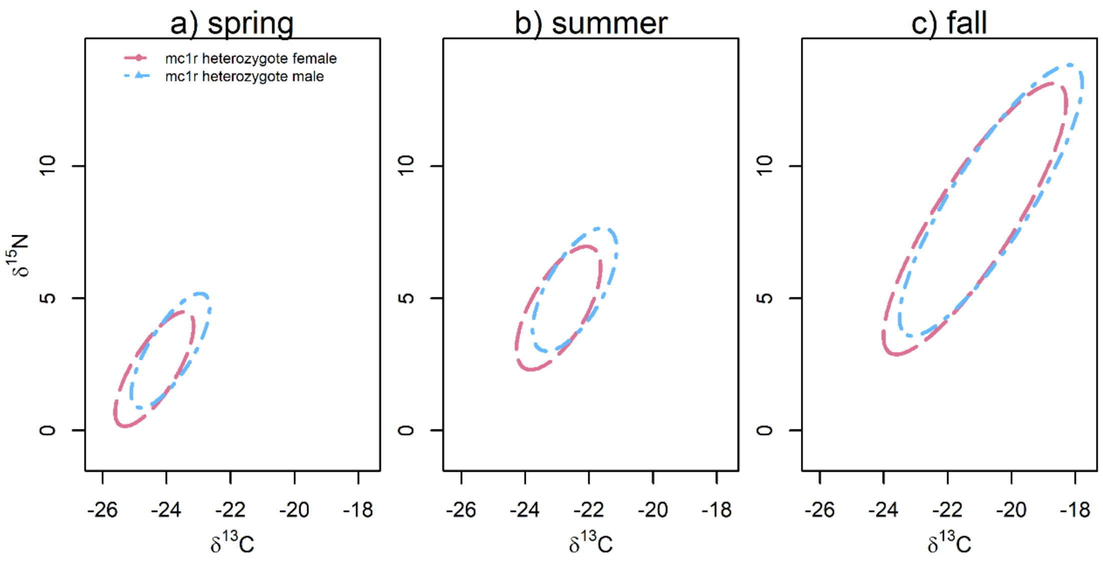

Figure S2. Seasonal isotopic foraging niche variation ($\delta^{13}C$ and $\delta^{15}N$) in coastal black bears (*Ursus americanus kermodei*) between male and female heterozygote (AG) *mc1r* genotypes (n = 6 female, n = 24 male) during a) spring (tip hair segment), b) summer (mid segment) and c) fall (base segment). Ellipses represent core niche area as inferred by multivariate repeated measures Bayesian linear mixed effect models.
